# Sinonasal Cholesteatoma Presenting Within the Maxillary Sinus: A Case Report

**DOI:** 10.1155/crot/9439954

**Published:** 2026-04-28

**Authors:** Emily Kwon, Avigeet Gupta, Liudmila Bardonova, Rachel Kaye, Andrey Filimonov, Wayne D. Hsueh

**Affiliations:** ^1^ Department of Otolaryngology-Head and Neck Surgery, Rutgers New Jersey Medical School, Newark, New Jersey, USA, rutgers.edu; ^2^ Department of Pathology, Cooperman Barnabas Medical Center, Livingston, New Jersey, USA

**Keywords:** case report, cholesteatoma, endoscopic sinus surgery, epidermal inclusion cyst, epidermoid cyst, maxillary sinus, paranasal sinus

## Abstract

**Objectives:**

Epidermal inclusion cysts are a common type of benign cutaneous cyst. When located in the mastoid cavity, they are more commonly known as cholesteatomas. However, it is extremely rare to be present within the paranasal sinuses, with 20 known cases involving the maxillary sinus. This is a case presentation of an epidermal inclusion cyst of the maxillary sinus, describing its clinical presentation, identifying pathological correlations, and highlighting the importance of early recognition and management.

**Study Design:**

A case report.

**Methods:**

A retrospective review of the medical records and pathology findings was performed.

**Results:**

A previously healthy 20‐year‐old male presented with several months of bilateral nasal obstruction refractory to medical therapy. Nasal endoscopy demonstrated a deviated septum and edema of the left middle meatus. Noncontrast computed tomography demonstrated opacification of the left maxillary sinus with bony expansion. Of note, there was the appearance of an ectopic tooth within the posterior aspect of the maxillary sinus. Surgical management included septoplasty and left endoscopic maxillary mega‐antrostomy. Histopathologic examination of the left maxillary sinus contents was diagnostic of an epidermal inclusion cyst. There was improvement of symptoms and no recurrence or residual cyst during postoperative follow‐up at 6 months.

**Conclusion:**

This case underscores the need to consider the extremely rare entity of an epidermal inclusion cyst within the differential diagnosis for chronic sinusitis and the need for careful review of imaging for surgical planning. Awareness of this presentation will facilitate accurate diagnosis and appropriate management of this rare disease.

## 1. Introduction

Cholesteatomas, or epidermal inclusion cysts, are benign aggregations of keratinized squamous debris that commonly present in the middle ear and rarely within the sinonasal cavity [1]. To the best of our knowledge, the English‐language literature contains only 52 reported cases of paranasal cholesteatoma, 20 of which originated from the maxillary sinus [1, 2]. They often present with nonspecific symptoms, such as nasal obstruction and purulent rhinorrhea, making the preoperative diagnosis challenging and resulting in frequent misdiagnosis for odontogenic sinusitis or allergic fungal sinusitis [2]. This case presentation highlights the rarity of this entity, particularly with odontogenic involvement.

## 2. Case Report

A previously healthy 20‐year‐old male presented to the outpatient otolaryngology clinic with several months of bilateral nasal obstruction refractory to medical therapy. Prior courses of antibiotics, steroids, and allergy medications provided only temporary relief. He denied any facial pain, pressure, hyposmia, or anosmia. He endorsed rhinorrhea with postnasal drip with mucopurulent discharge. The patient’s medical and surgical history was unremarkable.

**FIGURE 1:**
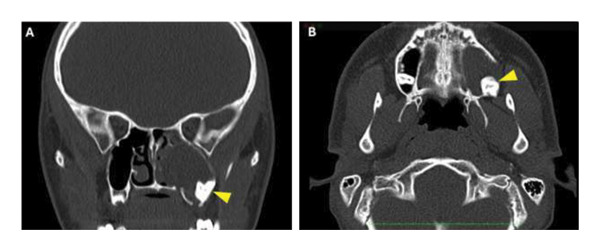
Computed tomography scans of case presentation. (A) Coronal view showing opacification of the left maxillary sinus with bony expansion, left ethmoid opacification, and odontogenic involvement (yellow arrow). (B) Axial view of left maxillary sinus with odontogenic involvement (yellow arrow).

**FIGURE 3:**
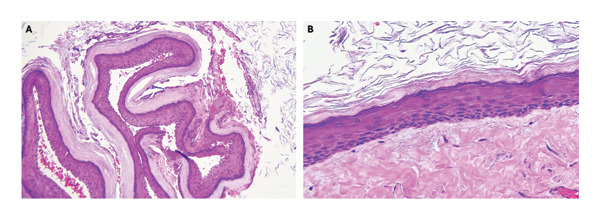
Histopathological images. (A) Microscopic exam reveals an epidermal inclusion cyst (hematoxylin and eosin (H&E) ×100). (B) A representative section of the cyst wall consists of stratified squamous epithelium with a distinct granular layer. Lamellated keratinous material and debris are present in the cyst lumen (H&E ×400).

Diagnostic rigid nasal endoscopy demonstrated a deviated septum and mucosal edema of the left middle meatus. Noncontrast computed tomography demonstrated opacification of the left maxillary sinus with bony expansion (Figures 1(A) and 1(B)). Of note, there was the appearance of an ectopic tooth posterior to the maxillary sinus opacification. This initially led to the presumed diagnosis of an odontogenic sinusitis. Surgical management included septoplasty and left endoscopic maxillary mega‐antrostomy (Figures 2(a), 2(b), and 2(c)). Histopathologic examination of the left maxillary sinus contents was diagnostic of an epidermal inclusion cyst after careful review by the pathologist. A microscopic exam revealed an epidermal inclusion cyst lined by stratified squamous epithelium, and lamellated keratinous material and debris were present in the cyst lumen (Figures 3(A) and 3(B)). At 6 months following surgery, patient reported improvement of symptoms. Exam on bilateral rigid nasal endoscopy showed patent sinuses and a tooth embedded on the posterior maxillary sinus wall. There was no recurrence or residual cyst observed during the postoperative follow‐up.

FIGURE 2Operative findings during left endoscopic maxillary mega‐antrostomy and total ethmoidectomy procedure. (a) Bulging of the left lateral nasal wall and edema of the middle meatus. (b) Keratin debris within the maxillary sinus cavity. (c) Keratin debris and tooth (yellow arrow) visualized embedded on the posterior maxillary sinus wall.

**Figure:**
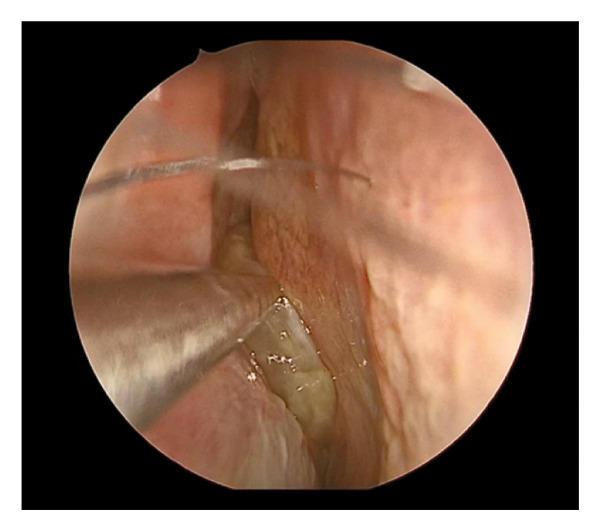
(a)

**Figure:**
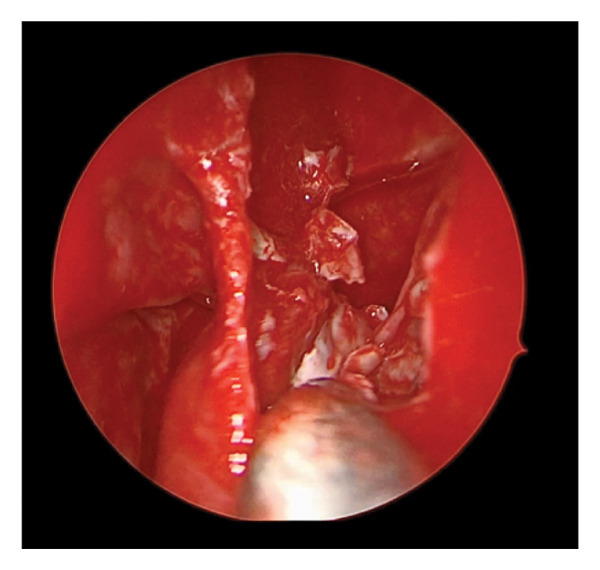
(b)

**Figure:**
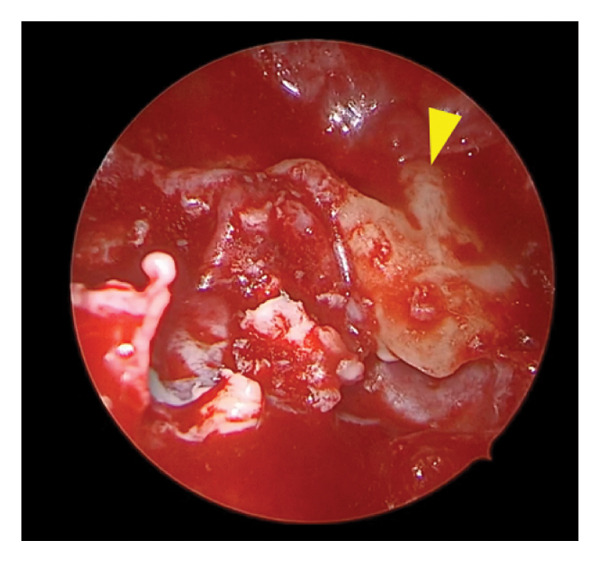
(c)

## 3. Discussion

Cholesteatomas of the maxillary sinus are rare, benign lesions characterized by the presence of keratinizing squamous epithelium within an ectopic location. Commonly known as a middle ear pathology, cholesteatomas rarely present in the paranasal sinuses [1]. A recent narrative review identified 51 cases of cholesteatoma involving the nasal cavity and paranasal sinuses reported between 1930 and 2024 [1]. Only 20 of these cases involved the maxillary sinus [1]. The differential diagnosis for unilateral sinusitis is broad, and includes mucoceles, odontogenic cysts, inverted papillomas, fungal balls, pyoceles, cholesterol granulomas, sebaceous cysts, and neoplasms (benign and malignant) [3]. Early recognition and differentiation from other sinonasal pathologies is vital due to the potential for local aggressive behavior, including bone erosion and extension into anatomically significant regions such as the orbit and anterior skull base [1].

We report a case of nasal sinus cholesteatoma arising within the maxillary sinus. Among reported cases of nasal and maxillary sinus cholesteatoma, a male predominance has been observed [1]. The age range in affected males is broad, spanning from 10 to 76 years; however, the condition is more commonly observed in middle‐aged and older individuals, making our patient’s presentation relatively uncommon [1]. Maxillary sinus cholesteatoma typically manifests as a slowly expanding lesion that may cause nasal obstruction, recurrent sinusitis refractory to medical management, dental loosening, check or hard palate swelling, and orbital complications, which are attributable to the close anatomical proximity of the maxillary sinus to the orbit and dental roots [1, 3]. Our patient presented with only several months of bilateral nasal obstruction refractory to medical therapy.

Several theories have been proposed to explain the pathogenesis of paranasal sinus cholesteatoma. The first suggests an origin from remnants of ectodermal epithelial cells during face development in the third to fifth week of gestation [4]. The second proposes that epithelial entrapment resulting from repeated episodes of inflammation, or disruption from prior surgical intervention, can displace squamous epithelium into ectopic locations [1]. A third theory holds that chronic inflammation may induce epithelial metaplasia or dysplasia, raising the possibility of neoplastic transformation in some cases [1]. In our patient, the absence of known trauma or prior sinus surgery raises the possibility of a congenital origin [4, 5]. The presence of his ectopic tooth also raises the likelihood that epidermal cells may have traveled with the tooth during development leading to the presence of the cholesteatoma.

The current case highlights a maxillary sinus cholesteatoma that was initially misdiagnosed preoperatively. Preoperative clinical diagnoses are challenging, as imaging may not differentiate cholesteatomas from other pathologies, necessitating the importance of histopathology following surgical intervention [6, 7]. The diagnosis was first suspected intraoperatively, based on the presence of keratin debris, and was subsequently confirmed through histopathologic examination.

Nasal endoscopy remains the first‐line diagnostic tool, offering direct visualization of mucosal abnormalities, as observed in our patient. Computed tomography is valuable for evaluating the extent of disease and bony remodeling, while diffusion‐weighted imaging (DWI) magnetic resonance imaging (MRI) can aid in differentiating paranasal sinus cholesteatoma from other sinonasal pathologies [1]. On MRI, cholesteatomas demonstrate no enhancement on T1‐weighted postcontrast sequences and exhibit high signal intensity on DWI, whereas nasal mucoceles typically show low DWI signal. DWI is particularly effective in identifying cholesteatomas as small as 3‐4 mm. Furthermore, MRI can help distinguish inflammatory sinus disease from sinus neoplasms [6, 8].

Definitive treatment for cholesteatomas requires complete wide surgical resection [9]. In our case, a maxillary mega‐antrostomy via an endoscopic approach allowed for complete excision of the lesion.

While cholesteatomas are benign, rare cases of malignant transformation have been documented [10–12]. Notably, four instances of squamous cell carcinoma arising from frontal sinus cholesteatomas and one case involving the maxillary sinus have been reported in the literature [10]. These findings underscore the importance of thorough histopathologic evaluation and long‐term surveillance, particularly in cases with incomplete excision or recurrence.

## 4. Conclusion

This case highlights the importance of including the extremely rare entity of an epidermal inclusion cyst in the differential diagnosis of unilateral maxillary sinusitis. These lesions can mimic more common sinonasal conditions, and delayed recognition may lead to complications if left untreated. Awareness of this potential diagnosis, particularly in patients with persistent unilateral nasal obstruction unresponsive to medical therapy—and in the absence of polyps or overt infection—can facilitate appropriate imaging review, surgical planning, and definitive treatment. In cases of refractory unilateral nasal congestion without a clear etiology, clinicians should maintain a broad differential and consider rare pathologies like epidermal inclusion cysts, especially when imaging reveals sinus opacification and bony changes.

## Funding

The authors have nothing to report.

## Conflicts of Interest

The authors declare no conflicts of interest.

## Data Availability

The data that support the findings of this study are available from the corresponding author upon reasonable request.
